# Additional evidence to support OCT-4 positive VSELs and EnSCs as the elusive tissue-resident stem/progenitor cells in adult mice uterus

**DOI:** 10.1186/s13287-022-02703-8

**Published:** 2022-02-05

**Authors:** Pushpa Singh, Siddhanath Metkari, Deepa Bhartiya

**Affiliations:** Stem Cell Biology Department, ICMR-National Institute for Research in Reproductive Health, Jehangir Merwanji Street, Parel, Mumbai, 400 012 India

**Keywords:** Endometrium, Regeneration, Endometrial scratching, Endocrine disruption, Stem cells, VSELs, EnSCs

## Abstract

**Objective:**

True identity and specific set of markers to enrich endometrial stem cells still remains elusive. Present study was undertaken to further substantiate that very small embryonic-like stem cells (VSELs) are the true and elusive stem cells in adult mice endometrium.

**Methods:**

This was achieved by undertaking three sets of experiments. Firstly, SSEA-1+ and *Oct-4* + positive VSELs, sorted from GFP mice, were transplanted into the uterine horns of wild-type Swiss mice and GFP uptake was studied within the same estrus cycle. Secondly, uterine lumen was scratched surgically and OCT-4 expressing stem/progenitor cells were studied at the site of injury after 24–72 h. Thirdly, OCT-4  expression was studied in the endometrium and myometrium of adult mice after neonatal exposure to estradiol (20 µg/pup/day on days 5–7 after birth).

**Results:**

GFP + ve VSELs expressing SSEA-1 and *Oct-4* engrafted and differentiated into the epithelial cells lining the lumen as well as the glands during the estrus stage when maximum remodeling occurs. Mechanical scratching activated tissue-resident, nuclear OCT-4 positive VSELs and slightly bigger ‘progenitors’ endometrial stem cells (EnSCs, cytoplasmic OCT-4) which underwent clonal expansion and further differentiated into luminal and glandular epithelial cells. Neonatal exposure to endocrine disruption resulted in increased numbers of OCT-4 positive VSELs/EnSCs in adult endometrium.

**Discussion:**

Results support the presence of functionally active VSELs in adult endometrium. VSELs self-renew and give rise to EnSCs that further differentiate into epithelial cells under normal physiological conditions. Also, VSELs are vulnerable to endocrine insults. To conclude VSELs are true and elusive uterine stem cells that maintain life-long uterine homeostasis and their dysregulation may result in various pathologies.

**Graphical Abstract:**

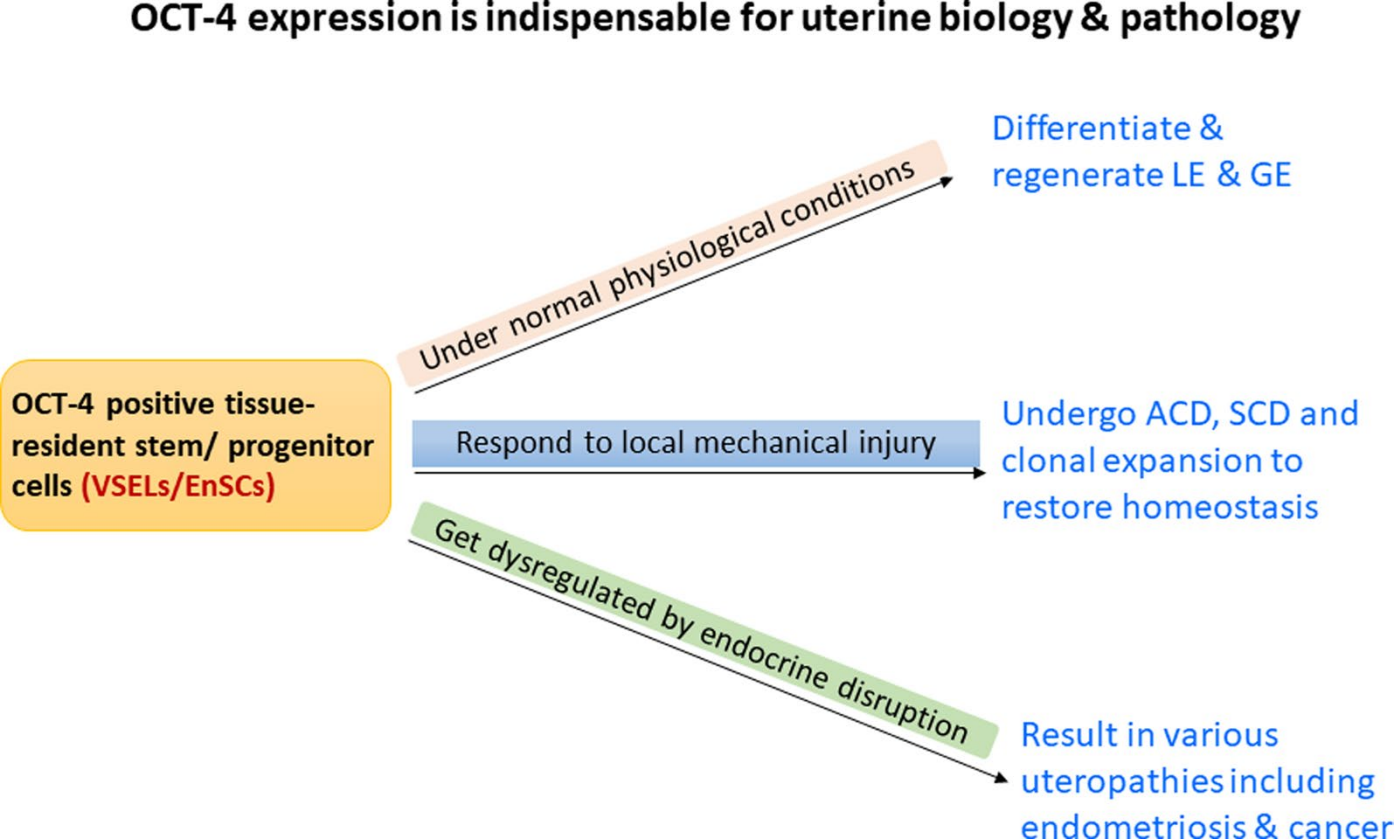

**Supplementary Information:**

The online version contains supplementary material available at 10.1186/s13287-022-02703-8.

## Background

Several groups are working towards identifying stem cells which ensure regeneration and growth of endometrium on regular basis across estrus/menstrual cycle and during pregnancy. However, set of markers to identify and enrich uterine stem cells as a physical entity remains elusive till date [1, 2]. Bone marrow-derived cells have been suggested to have a role in endometrial and decidual physiology as well as the pathophysiology of endometriosis [3, 4] but remain controversial [5], others have advocated mesenchymal stem cells (MSCs) as possible uterine stem cells [6, 7] while recent lineage tracing studies reported presence of bipotent Keratin-19 [8] and AXIN-2 [9, 10] positive epithelial stem/progenitor cells. Our group has reported two populations of OCT-4 positive stem/progenitor cells including nuclear OCT-4 positive very small embryonic-like stem cells (VSELs) and progenitors termed endometrial stem cells (EnSCs) with cytoplasmic OCT-4 that exist in both endometrium and myometrium [11–14]. VSELs/EnSCs express receptors specific for estradiol (E), progesterone (P) and follicle stimulating hormone (FSH), show distinct changes across estrus cycle and respond to E, P and FSH treatment in both the endometrium and myometrium [13, 14]. VSELs are developmentally equivalent to the primordial germ cells and have been suggested to migrate to all the developing organs in the body during early development and survive throughout life [15]. They maintain tissue homeostasis and their existence in multiple adult tissues has been confirmed by 50 independent groups [15]. Uterine VSELs/EnSCs are affected by neonatal exposure to endocrine disruption, increase in numbers and their altered functions could lead to initiating various uteropathies [16]. Highlights of our results so far on uterine VSELs/EnSCs are listed as Additional file 1: Table S1. However, there is no consensus in the scientific community on the specific markers to identify uterine stem cells and complete disbelief exists in the presence of VSELs/EnSCs in adult mice uterus.

MSCs have no role in regenerating the luminal (LE) and glandular (GE) epithelial cells based on an in vivo cell fate tracing study [9, 17]. Jin [8] used CreERT2-LoxP–based single-cell lineage tracing system in the adult mouse uterus to functionally identify epithelial stem cells. In this study, a mouse cell line containing Keratin-19 (epithelial marker) promotor positioned next to an inducible Cre recombinase was crossed with the Rosa26-YFP reporter strain to lineage label epithelial cells. Bipotent endometrial epithelial stem cells were detected which differentiated into LE and GE for maintaining homeostasis and regeneration of mouse endometrial epithelium under physiological conditions [8]. Another lineage-tracing study reported Axin2-expressing cells residing in endometrial glands as the possible stem cells responsible for epithelial regeneration [9]. Here tetracycline induction and Cre-mediated recombination system were combined to label and trace the behavior and fate of the Axin2-expressing cells. These cells were detected at the base of the glands and expanded to occupy the whole gland over a period of 70–90 days. Cousins et al. [1] argued that this period of 70–90 days being equivalent to 22–23 estrus cycles (each comprising 4–5 days) and thus contribution of Axin2 positive cells was very limited in nature. Even during the studies to track these cells after six cycles of pregnancy and involution, their contribution towards LE remained limited and as a result Axin2 possibly remains a marker for GE progenitor cells [1, 9]. Cellular turnover of LE is much higher compared to GE in cycling mice [18]. Other markers including Foxa2c, Lgr5c and Pax8c were also used for lineage tracing studies but have not been very helpful in identifying the epithelial stem/progenitor cells. Lineage tracing of Foxa2C GE fate completely excluded the contribution of GE to LE [8]. Lgr5C progenitor cells are exclusively responsible for the development and maintenance of uterine glands [19]. Pax8 epithelial cells could maintain both LE and GE, however, it is difficult to track Pax8 positive cells as they are abundant throughout the entire endometrial epithelium [20].

Standard methods that are generally used to study stem cells hierarchy in adult tissues were recently compiled by Cousins et al. [1] and Miguel-Gomez et al. [2]. These methods include approaches like in vitro functional assays (clonogenicity assays, proliferation assays, evaluating differentiation potential), side population, label retaining cells and most recent and now considered a gold-standard is lineage tracing studies. Possibly because of this mindset to study stem cells, our work on uterine VSELs/EnSCs does not even get noticed and cited. None of the above-mentioned conventional and gold standard approaches were used by us to report the presence of VSELs/EnSCs in the uterus. Being quiescent in nature, VSELs will never get labeled and detected during lineage tracing studies. Their small size and scarce nature lead to their inadvertent loss while processing and thus do not get detected during flow cytometry/side population and scRNAseq studies [21, 22]. It is important to appreciate these limitations with available tools to study ‘quiescent’ and ‘rare population’ of stem cells. It is possibly because of these novel attributes that uterine VSELs have remained elusive till date.

Present study provides further crucial evidences to authenticate that indeed OCT-4 positive VSELs are the elusive, tissue-resident uterine stem cells that can differentiate into both LE and GE in an efficient manner under physiological conditions and get dysregulated upon neonatal exposure to endocrine disruption. Firstly, role (if any) of SSEA-1 sorted VSELs from FVB-GFP mice was studied upon transplantation into the mice uterus under physiological conditions. Secondly, effect of endometrial scratching was studied on OCT-4 expression within 24–72 h. Thirdly, OCT-4 expressing stem/progenitor cells were studied in the adult mice endometrium after neonatal exposure of pups to estradiol.

## Study design and methods

### Study design

FVB-GFP (constitutively express GFP) and Swiss mice, maintained in the Institute Experimental Animal Facility were used for the present study. The animals were housed under controlled temperature of (23 ± 1 °C) and humidity (55 ± 5%), with 14 h light and 10 h dark cycle with free access to food and water. All experiments carried out in the present study were approved by NIRRH Institutional Animal Ethics Committee (78/GO/ReBi/SL/99/CPCSEA dated-11/03/1999).

### Tracking differentiation potential of GFP + SSEA-1 + Oct-4A + VSELs upon uterine transplantation

SSEA-1 positive VSELs were isolated during estrus stage (since VSELs are maximal in numbers at this stage) from FVB-GFP mice uterus (*n* = 8) by immuno-magnetic isolation using fluorescein isothiocyanate tagged SSEA-1 antibody (BD Biosciences, San Diego, California) and EasySep Kit (Stem Cell Technology, Vancouver, Canada) as described earlier [23]. Care was taken to pellet cells by centrifuging at 1000*g* for 15 min. Approximately 1 million cells were transplanted into both the horns of two months old Swiss mice uterus during diestrus when the uterus is maximally regressed. After two days of rest, mice were checked for cyclicity and were sacrificed during estrus. The uterine tissue was fixed in 4% paraformaldehyde and processed using standard methods to make paraffin blocks. The transplanted cells were tracked for GFP expression by immuno-fluorescence and confocal microscopy. Our approach enabled selective tracking of GFP + transplanted, SSEA-1 + cells with no overlap with tissue-resident GFP- and SSEA-1 + stem cells. Sorted SSEA-1 positive cells were also placed in TRIzol for RNA extraction to study *Oct-4A* and *Oct-4* expression.

### OCT-4 + VSELs/EnSCs are activated and initiate endometrial regeneration in response to mechanical injury

We hypothesized that uterine scratching would inflict injury that may activate the tissue-resident stem cells. Mechanical injury was inflicted to the endometrium during diestrus stage in adult mice uterus. For this, the uterus was exposed by an excision in the lower mid-line abdomen. 16G needle was inserted into the uterine body (connection between the left and right horns) and both the horns were scratched back and forth gently until they became hypermic to the naked eye as described in literature [24]. The mice were sacrificed after 24 and 72 h, fixed in paraformaldehyde, processed and paraffin blocks were prepared to study histology and OCT-4 expression.

### Increased OCT-4 expression in endometrium of adult mice which were neonatally subjected to endocrine disruption

Mice pups were exposed to estradiol on postnatal days 5–7 (20 µg/pup/day) as described recently [16] and then uterine tissue was collected on D100 during adult life for immuno-localization of OCT-4 positive cells in endometrial sections.

## Materials and methods

### Enrichment of uterine stem cells

This has been described in details earlier [14]. In brief, uterine horns of FVB-GFP mice were collected in Dulbecco phosphate buffer saline (DPBS) and after 3 washes, chopped and subjected to enzymatic digestion with collagenase type IV (Gibco Life Technologies, 1 mg/mL) in Dulbecco modified Eagle medium (DMEM/F12, Gibco) at 37 °C for 20 min with intermittent shaking. Trypsin EDTA (Gibco, 0.25%) was added for additional 10 min at 37 °C to obtain a single-cell suspension. 20% fetal bovine serum (FBS, Gibco) was added to inactivate trypsin and the cells suspension was filtered through 70 and 40 µm cell strainers (Corning). Cells pellets collected after centrifuging at 1000*g* for 15 min (Biofuge Stratos, Heraeus) were subjected to red blood cells lyses (if required) using 1X RBC lysis buffer (hypotonic ammonium chloride solution) for 10 min. The cells were re-suspended in DPBS and spun at 250 g to collect bigger somatic cells (Pellet A). Supernatant was further spun at 1000 g to enrich stem cells (Pellet B).

### Immuno-magnetic isolation of SSEA-1 positive cells

SSEA-1 + stem cells were isolated from pellet B enriched for stem cells (described above) by immuno-magnetic method (Stem Cell Technologies). In brief, cells in pellet B were resuspended in 250 µl EasySep buffer in a round bottom flow-tube and FCR blocker was added for 10 min to remove any non-specific binding. After that SSEA-1 antibody (PE conjugated, BD Bioscience) was added, mixed well by pipetting and incubated for 15 min followed by PE selection cocktail for 15 min. Later nanoparticles were added for 10 min according to manufacture instructions. Then 2.5 ml cells suspension was passed through a column within a magnetic field. Cells carrying the magnetic beads were retained inside the column, which attracted even slightly magnetized cells onto the column surface. The unbound SSEA-1 negative cells  were washed away. This step was repeated twice by adding fresh EasySep buffer. The beads-carrying cells were recovered by elution after turning off the magnetic field and cells were centrifuged at 1000*g* for 10 min. These cells were counted and used for transplantation.

### Immuno-localization of GFP and OCT-4

Briefly, the paraffin embedded uterine tissue sections were deparaffinized by placing in xylene for 15–20 min twice. After air drying, the sections on the slides were gradually hydrated in descending series of methanol and finally placed in tap water for 5 min. This was followed by antigen retrieval step by immersing the slides in boiling sodium citrate (SSC, Sigma) buffer at pH 6 for 10 min. After cooling, the slides were dipped in water followed by 1X PBS for 5 min each. Blocking was done with 5% normal goat serum plus 1% BSA for 1 h followed by overnight incubation with the primary antibody (1:100, GFP ABCAM ab290, OCT-4 Millipore MAB4419) at 4 °C. Primary antibody was omitted and replaced with blocking solution to serve as the negative control. Next day slides were washed 3 times with PBS (5 min each wash) and then incubated with Alexaflour 488/568 anti-rabbit/anti mice 1:500 dilutions (Invitrogen Life Technologies) raised in goat for 2 h, followed by 3 washes with PBS and then counterstained with DAPI (4′,6-diamidino-2-phenylindole; 1:200; Invitrogen) for 30 min. After 2 PBS washes, the slides were mounted with Vectashield (Vector laboratories, CA 94010) and viewed under Olympus (Fluoview Fv 3000). Representative images were taken. Negative controls with omission of primary antibody were included in the experiments.

### RT-PCR and qRT-PCR studies

SSEA-1 and GFP positive cells were studied for *Oct-4A* and *Oct-4* expression by RT-PCR in the first experiment. qRT-PCR studies were done to study differential expression of *Oct-4A, Oct-4 and Sca-1* at 24 and 72 h after mechanical scratching. Primers used were for *Oct-4* (annealing temperature 61 °C, product size bp177): F: CCTGGGCGTTCTCTTTGGAAAGGTG, R: GCCTGCACCAGGGTCTCCGA; *Oct-4A* (annealing temperature 62^0^C, product size bp235): F: CCATGTCCGCCCGCATACGA, R: GGGCTTTCATGT CCTGGGACTCCT; *Sca-1* (annealing temperature 60^0^C, product size bp223): F: AGAGGAAGTTTTATCTGTGCAGCCC, R: TCCACAATAACTGCTGCCTCCTGA; *18S* (annealing temperature 60 °C, product size bp171): F: GGAGAGGGAGCCTGAGAAAC, R: CCTCCAATGGATCCTCGTTA.

#### RNA extraction and cDNA preparation

RNA was isolated from SSEA-1 positive cells and uterine tissue after 24 and 72 h of uterine scratching which were stored in TRIzol (#15596018, Genex Life Sciences) using manufacturer’s instructions. First-strand cDNA was synthesized using the iScript cDNA synthesis Kit (#1708891, Bio-Rad, USA) according to the manufacturer’s instructions.

#### RT-PCR

Briefly, 1 µg of total RNA was incubated with 5 × iScript reaction mix and reverse transcriptase mix. The reaction was carried out in G-STORM thermocycler (Gene Technologies, UK) as per manufacturer’s instructions. RT-PCR was carried out to detect the expression of pluripotent transcripts (*Oct-4, Oct-4a).* The cDNA mix (2 μl) was amplified using 0.2 mM of each primer, 1.25 unit of DreamTaq DNA polymerase (Fermentas) in 1X buffer and 0.2 mM dNTPs in a G-STORM thermocycler. Amplification was carried out for 35 cycles, with each cycle consisting of denaturation at 94 °C for 30 secs, annealing at the specified temperature for 20 secs, and extension at 72 °C for 30 s. The products were analyzed on 2% agarose gel stained with 0.5 μg/ml ethidium bromide (Bangalore Genie). The product size was approximated using a 100 bp DNA ladder (Bangalore Genie).

#### Quantitative RT-PCR

qRT-PCR analysis was done in CFX96 real-time PCR system (Bio-Rad Laboratories, USA) using SYBR Green chemistry (Bio-Rad). 18s was used as housekeeping gene. The amplification conditions were initial denaturation at 94 °C for 3 min followed by 40 cycles comprising of denaturation at 94 °C for 10 s, annealing for 20 s, and extension at 72 °C for 30 s. All PCR amplifications were carried out in duplicate. Mean Ct values generated in each experiment using the CFX Manager software (Bio-Rad) were used to calculate the mRNA expression levels. The fold change was calculated using ΔΔCt method.

## Results

### Tracking differentiation potential of GFP + SSEA-1 + Oct-4A + VSELs upon uterine transplantation

**Fig. 1:**
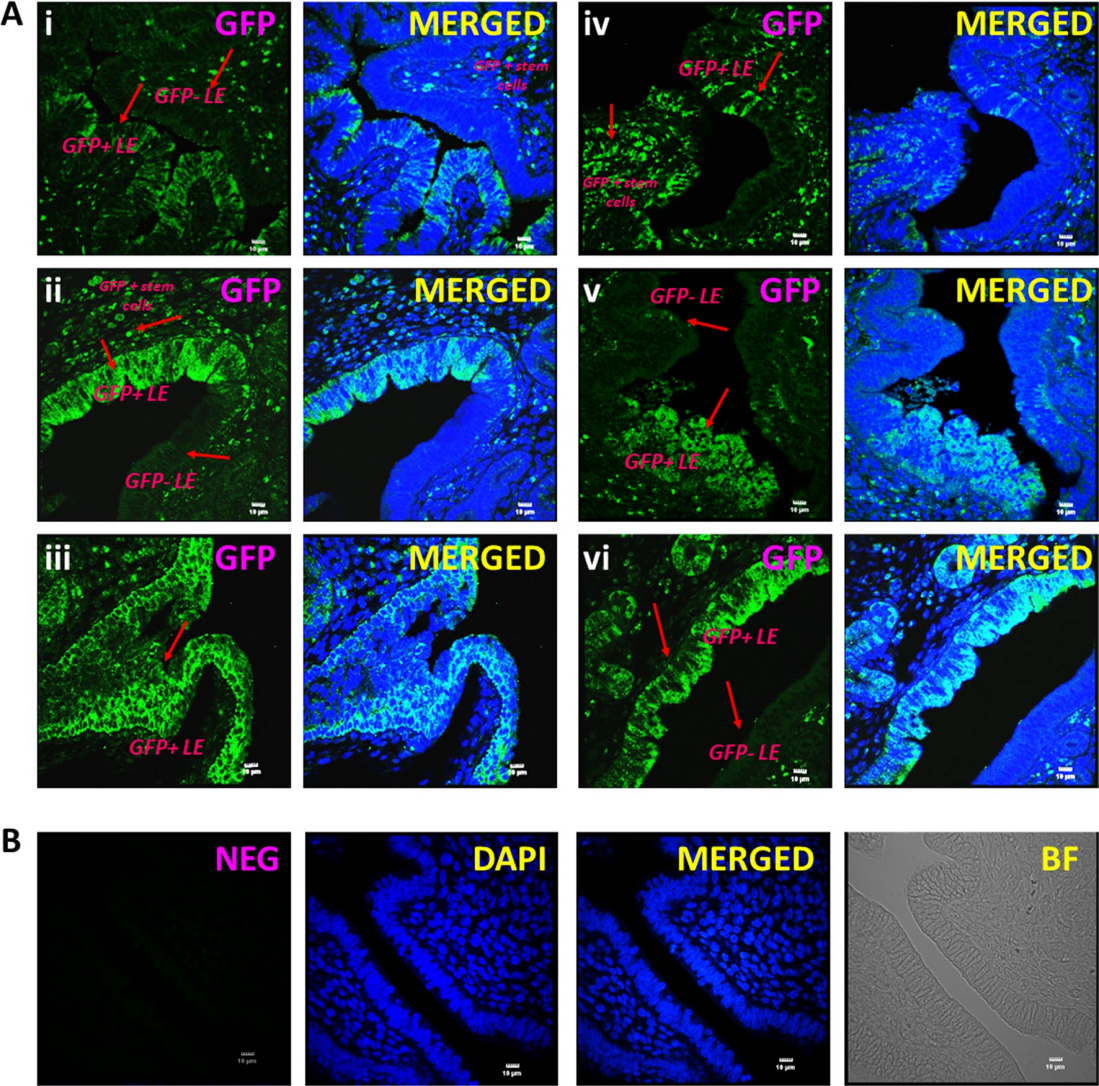
Engraftment of GFP + cells in the luminal epithelium. **A i–vi**. GFP positive cells were observed in the stroma and in the epithelial cells lining the luminal epithelium. Distinct mosaicism was evident in GFP expression with areas remaining negative for GFP suggesting that both transplanted and tissue-resident SSEA-1 and *Oct-4*A positive cells participated in endometrial regeneration. **B** Negative control. Primary GFP antibody was replaced with blocking solution to serve as the negative control. Scale:10 μm

As expected, RT-PCR results showed that GFP and SSEA-1positive stem cells expressed *Oct-4A* (Additional file 1: Fig. S1). These GFP + SSEA-1 + *Oct-4A* + stem cells, transplanted during diestrus stage of estrus cycle, were found to participate in uterine remodeling under physiological conditions during estrus stage of the same cycle. The estrus cycle was delayed 2 days due to the surgery but mice were sacrificed during estrus stage of the same cycle (4 days after transplantation). Transplanted, small-sized GFP positive cells were observed scattered in the stroma (Fig. 1). Interestingly these cells differentiated into LE and GE cells within the same cycle and distinct mosaicism was observed clearly showing GFP positive cells engraftment/differentiation into the luminal epithelium and the glands. Negative control with omission of primary GFP antibody showed no staining (Fig. 1B).

**Fig. 2:**
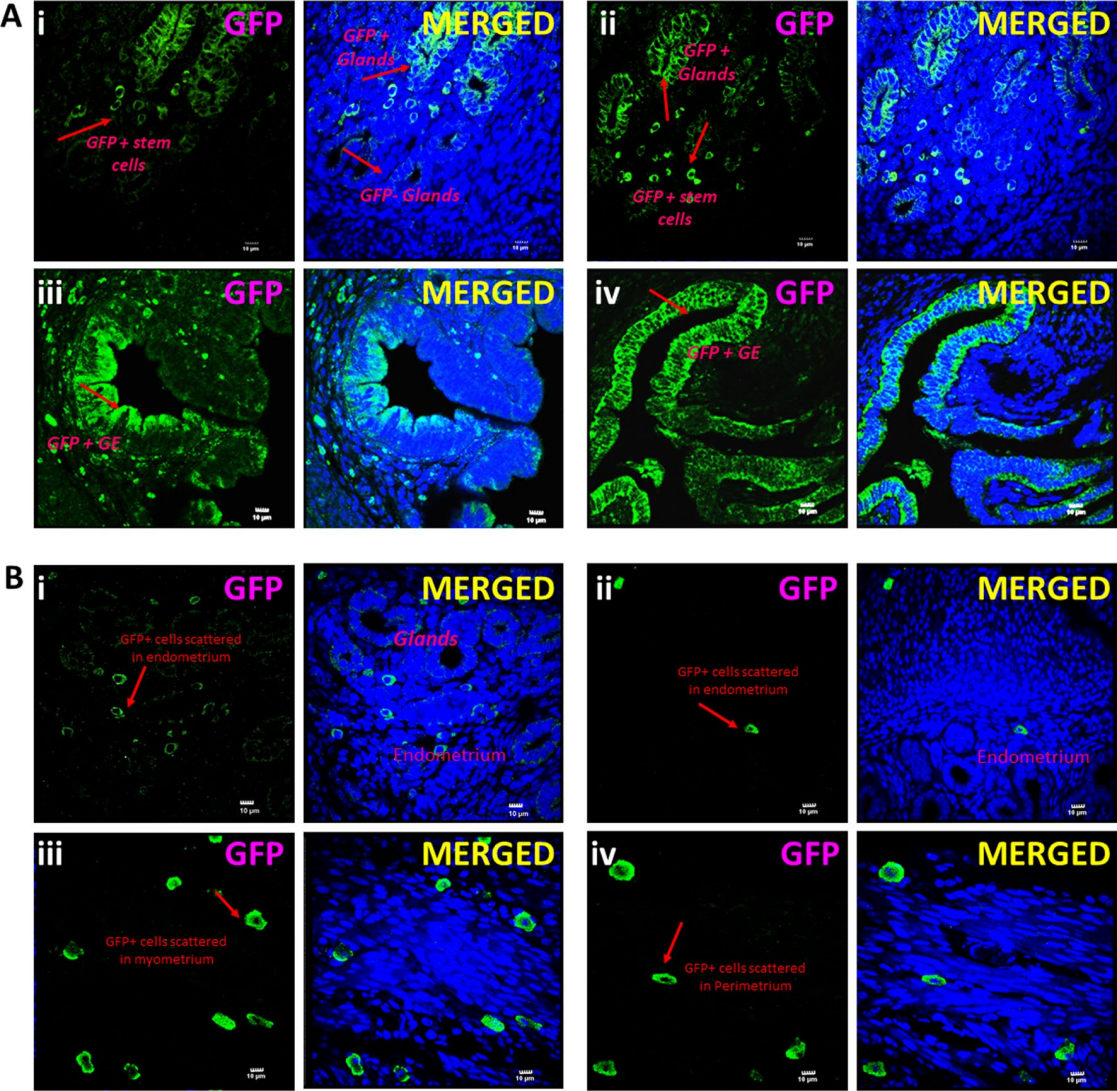
Engraftment of GFP positive cells in the glands. **A i–iv** GFP + cells were clearly observed in the stroma and amongst the epithelial cells lining luminal and glandular epithelium. GFP positive cells were incorporated in the glandular epithelium cells in few glands while few glands remained negative (**i–ii**,** iv**). Similarly, only a fraction of cells lining the luminal epithelium were GFP positive. **B i-iv** GFP positive bigger, differentiated epithelial and stromal cells in Pellet A did not engraft into the luminal and glandular epithelium. They were scattered in the stromal and myometrial compartment. Scale:10 μm

The GFP positive cells were clearly observed, nuclear GFP positive VSELs differentiated into epithelial cells with cytoplasmic GFP, and these cells also differentiated into GE cells. A mosaic pattern of native glands and those differentiating from GFP + cells was clearly evident (Fig. 2). It was clearly observed by the mosaic pattern of the glands with only a fraction of cells being GFP positive, that both tissue-resident and transplanted stem cells participated during remodeling and regeneration.

Transplanting GFP positive somatic cells (Pellet A, comprising of epithelial and stromal cells) into the uterus showed that these somatic cells remained scattered in the stromal and myometrial compartment and did not participate in endometrial regeneration (Fig. 2B).

### OCT-4 + VSELs/EnSCs get activated and initiate endometrial regeneration in response to mechanical injury

**Fig. 3:**
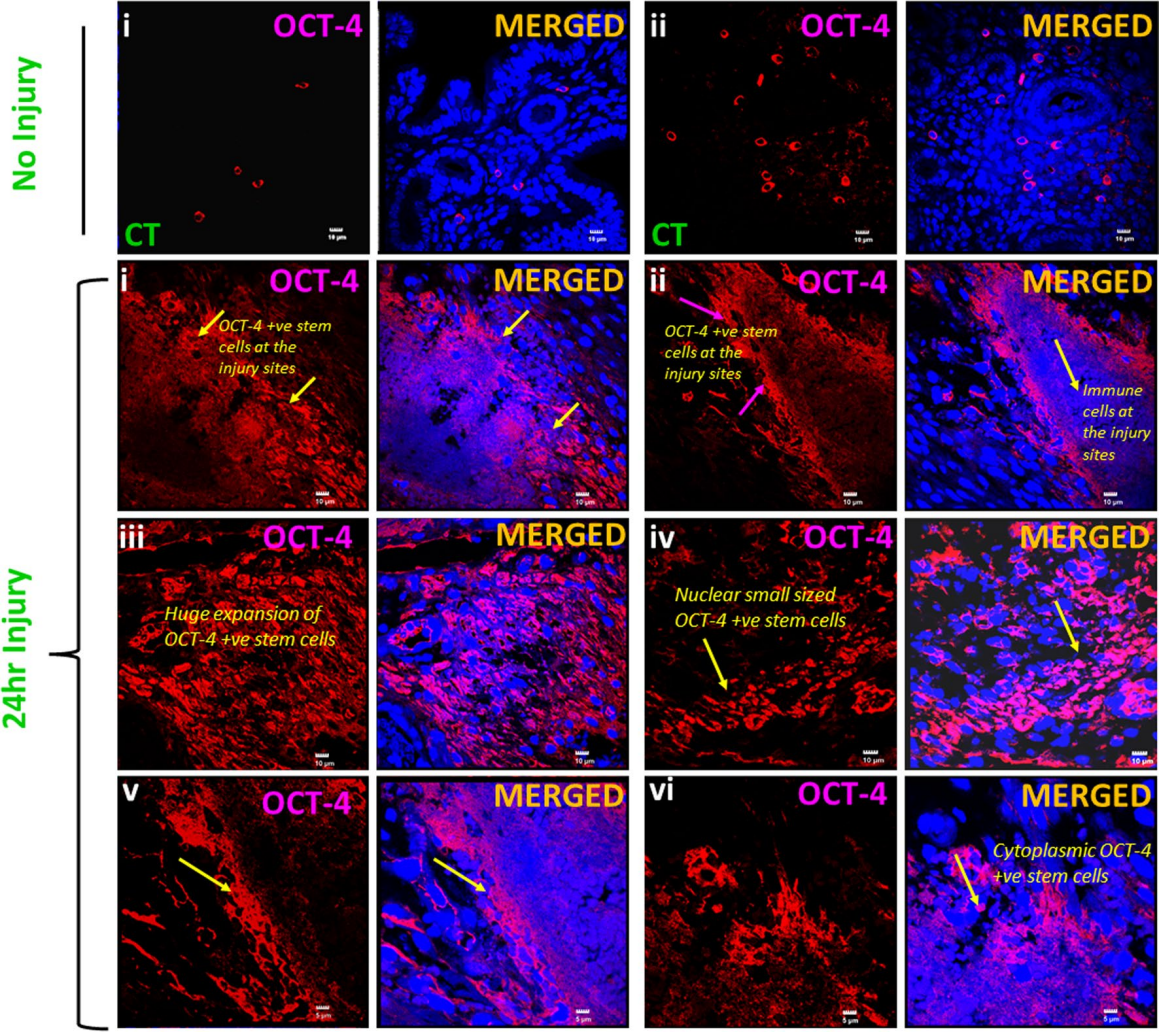
Expansion of OCT-4 positive stem cells upon endometrial scratching. Upper panel: OCT-4 expression in control uterus without any injury. OCT-4 positive progenitor cells were occasionally detected in the stromal and myometrial compartment. OCT-4 was not expressed by the mature differentiated epithelial and stromal cells. Lower panel shows increased expression of OCT-4 after 24 h of endometrial scratching. OCT-4 positive cells were activated in large numbers upon mechanically scratching the uterine lumen within 24 h. OCT-4 positive stem cells with both nuclear and cytoplasmic OCT-4 were clearly observed in large numbers in endometrial sections. Immune cells also accumulated at the injury site but remained negative for OCT-4 (**i**, **ii**, **v**, **vi**) while small-sized OCT-4 positive stem cells were observed in close vicinity at the injury site and helped to regenerate and maintain normal homeostasis (**i**–**vi**). OCT-4 positive stem cells came out from their quiescent state and expanded in large numbers to regenerate and regain homeostasis and wound healing. Scale: **i**–**iv**: 10 μm and **v**–**vi**: 5 μm

In this experiment, tissue-resident stem cells were expected to expand in response to injury induced by uterine scratching and participate during regeneration. In the normal control endometrium OCT-4 positive stem cells were mostly randomly scattered in the stromal and myometrial compartment and they were present in very less numbers (Fig. 3, Upper panel) while after endometrial scratching within 24 h we observed extensive expansion of small sized nuclear OCT-4 positive stem cells in the endometrium and were clearly visualized by immuno-fluorescence staining and confocal imaging (Fig. 3, Lower panel). Large numbers of cells were also accumulated at the injury sites and were surrounded by huge numbers of stem/progenitor cells. Also, histological study at 24 h showed the presence of large numbers of small, spherical, darkly stained cells with high *N*/*C* ratio in the basal region of LE in Hematoxylin and Eosin-stained sections (Additional file 1: Fig. S2). These putative stem cells were similar to those reported earlier wherein they were found to increase in numbers in bilaterally ovariectomized uterus upon treatment with estradiol, progesterone and FSH [13]. After 72 h, increased numbers of newly differentiated epithelial cells became evident (Additional file 1: Fig. S3), there was lot of proliferation and stem cells were also evident after 72 h along with PCNA expression (Additional file 1: Fig. S4–5).

**Fig. 4:**
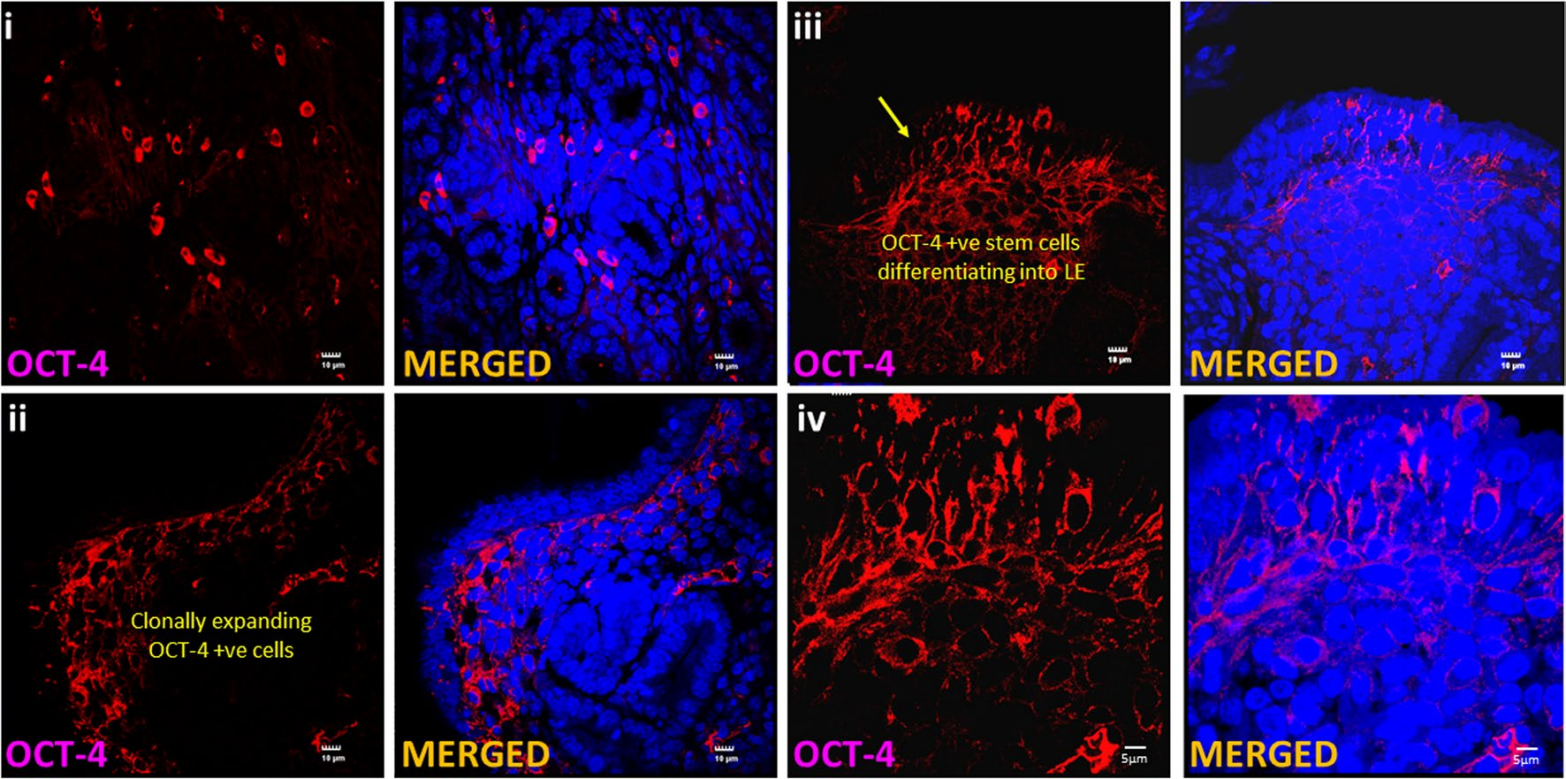
OCT-4 expressing stem cells expanded in numbers and differentiated into epithelial cells lining the LE. OCT-4 positive stem cells including both VSELs and EnSCs were activated in response to mechanical injury (**i**). After 72 h of uterine injury, large numbers of EnSCs with cytoplasmic OCT-4 became evident (**ii**). At places EnSCs were observed to undergo symmetrical cell divisions and clonal expansion (**iii**, **iv**). Evidently OCT-4 expressing stem/progenitor cells differentiate into epithelial cells and ensure widespread regeneration within 72 h. Scale: 10 μm (**i**–**iii**), 5 μm (**iv**)

After 72 h, cells expressing nuclear and cytoplasmic OCT-4 were found scattered in the endometrial stroma and at the base of the luminal epithelium (Fig. 4i, ii). Evidence to support differentiation of OCT-4 positive progenitors into LE cells is shown in Fig. 4iii–iv. Clonal expansion of OCT-4 positive cells was evident (Fig. 4ii).

OCT-4 positive stem cells contributed significantly towards endometrial regeneration within a short duration of 72 h. We have earlier reported asymmetrical, symmetrical divisions and clonal expansion of uterine VSELs and EnSCs [13, 25]. qRT-PCR results showed increased expression of *Oct-4A* and *Oct-4* specific to VSELs/EnSCs after 24 h, EnSCs were also increased in numbers after 72 h but VSELs were reduced as *Oct-4* was expressed but *Oct-4A* levels were reduced (Additional file 1: Fig. S6). Similarly, *Sca-1* expression was increased at 24 h and remained elevated after 72 h.

### Increased OCT-4 expression in endometrium of adult mice which were neonatally subjected to endocrine disruption

**Fig. 5:**
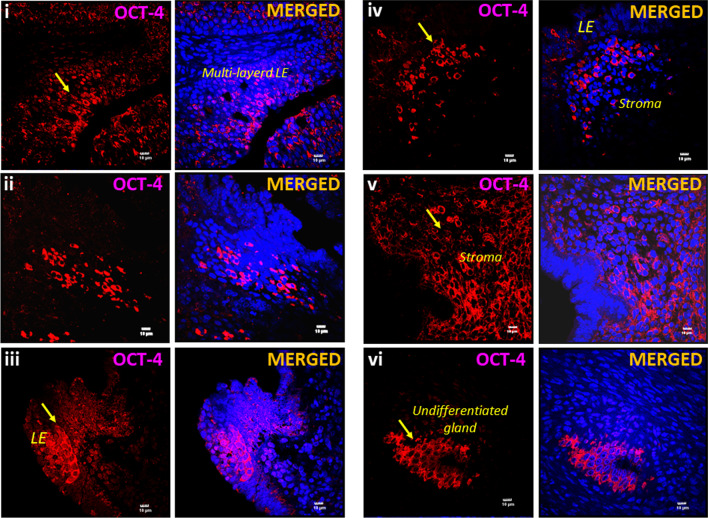
Expansion of endometrial  stem/progenitor cells compartment after neonatal exposure to endocrine disruption. OCT-4 positive stem/progenitor cells were present in large numbers in endometrial sections of adult, 100 days old mice endometrial sections after neonatal exposure to endocrine disruption. OCT-4 expression was observed in large numbers in luminal epithelium, stroma and immature undifferentiated glands in stromal compartment. Scale: 10 μm

Tissue resident stem cells/progenitors are directly vulnerable to endocrine disruption since they express receptors specific for ERα, ERβ and FSHR [14]. We recently reported increased numbers of stem cells by qRT-PCR (> 10 folds, > 4 folds *Sox-2* and > 5 folds *Nanog* in the Pellet B) and their blocked differentiation in adult uterus due to neonatal exposure to endocrine disruption [16]. These results were further confirmed in the present study since increased expression of OCT-4 positive VSELs was evident in uterine sections. Evidently, VSELs come out from their quiescent state due to neonatal exposure to endocrine disruption, undergo uncontrolled asymmetrical, symmetrical cell divisions and the progenitors EnSCs expand in large numbers (Fig. 5). While in control uterus, few OCT-4 positive stem/progenitors were randomly scattered and OCT-4 was not expressed by mature and well-differentiated epithelial cells (Fig. 3, upper panel).

OCT-4 positive stem/progenitor cells were observed in large numbers amongst the epithelial cells which showed hyperplasia (Fig. 5). The OCT-4 positive stem/progenitor cells were mobilized in large numbers in the stroma. Under normal conditions, OCT-4 expression is lost upon differentiation and is not observed in fully differentiated luminal and glandular epithelial cells (Fig. 3, upper panel). But abundant expression of OCT-4 in both luminal and glandular epithelium suggested a more primitive, undifferentiated state that results in various pathologies as discussed [16].

## Discussion

Present study provides additional evidence to support OCT-4 positive stem/progenitor cells participate in endometrial regeneration under normal physiological conditions and also after mechanical injury in adult mice. Transplanted GFP + SSEA-1 + *Oct-4* + VSELs (isolated from adult uterus) differentiated into the luminal and glandular epithelial cells and participated in regeneration along with the tissue-resident VSELs resulting in a mosaic pattern within 4 days in the same estrus cycle. Tissue-resident OCT-4 positive stem cells were activated and exhibited proliferation and clonal expansion to regenerate the endometrium after mechanical scratching. Also, OCT-4 positive stem/progenitor cells were increased in numbers as a result of neonatal exposure to endocrine disruption and were observed in abundance in the endometrium as well as in the myometrium which leads to various uteropathies [16].

We have reported presence of VSELs along with spermatogonial stem cells (SSCs) in testes [26] and ovarian stem cells (OSCs) in the ovaries [27]. Taking advantage of this knowledge and the fact that VSELs exist in all adult tissues in mice [15, 28, 29], we have studied and reported VSELs in both adult mice endometrium and myometrium and highlights of our published work are provided in Additional file 1: Table S1. Robust protocols are now available to study VSELs in testes [30], ovaries [31] and uterus [14]. Cell surface markers to study pluripotent VSELs include SSEA-1 and SCA-1 in mice and SSEA-4 and CD133 in humans.

Although there is a lack of consensus on the cell surface markers for human uterine stem cells, it is intriguing to point out that several groups have reported cell surface markers CD133 and ALDH-1 in side population enriched from human endometrial cancer tissues [32–34]. These markers are indeed specific for VSELs [35, 36] and although there is no report on VSELs in normal human uterine tissue, consensus seems to exist in stem cells markers in endometrial cancer possibly because of increased numbers of OCT-4 positive stem/progenitor cells in disease state. OCT-4 expression has been reported in human endometrium by few groups (Table 1) and it is interesting to point out that several groups have reported OCT-4 in eutopic and ectopic endometrium in ovarian endometriosis, endometrial hyperplasia and cancer where these cells exist in abundance. Similar pluripotent VSELs exist in bone marrow which get mobilized [15] and Taylor’s group has reported role of non-hematopoietic, bone marrow stem cells during endometrial regeneration, pregnancy and in endometriosis [37, 38]. Thus, it is data published by several independent groups that suggest involvement of OCT-4 positive stem/progenitor cells in endometrial biology.

**Table 1:** OCT-4 and other stem cell markers reported in human endometrium by other groups

Reference	Salient findings
Usta et al. (2020) PMID: 33218351	Reported differential expression of Oct-4, CD44, and E-cadherin in eutopic and ectopic endometrium in ovarian endometriomas
Anwar and Amer (2021)	Reported OCT4, Ki-67 and VEFF as prognostic factors in endometrial carcinoma and their role in the differentiation between atypical endometrial hyperplasia and grade 1 endometrial carcinoma. https://doi.org/10.21608/JCBR.2021.52448.1101
Shariati et al. (2019) PMID: 31417979	Increased expression of stemness genes REX-1, OCT-4, NANOG and SOX2 in women with ovarian endometriosis versus normal endometrium
Proestling et al. (2016) PMID: 27881125	Reported co-expression of OCT4 with stemness markers (SOX 15 and Twist 1) in epithelial and stromal cells of endometriotic samples
Pitynski et al. (2015) PMID: 26339387	Reported co-expression of nuclear OCT-4 and SOX2 in endometrial adeno-carcinoma tissue
Song et al. (2014) PMID: 24884521	Higher expression of Nanog and Sox2 along with lower OCT4 mRNA and higher OCT4 protein expression in ectopic endometrium by qRT-PCR, Western blotting and IHC
Chang et al. (2013) PMID: 23290742	Expression of OCT4 and NANOG mRNA was significantly higher in ectopic endometriotic tissues, compared with that of the normal endometrium, normal myometrium, and hyperplastic endometrium
Silveria et al. (2012) PMID: 22940770	Reported positive immunostaining for CD9, CD34, c-Kit and Oct-4 markers in isolated epithelial and/or stromal cells in eutopic and ectopic endometrium
Zhou et al. (2011) PMID: 21464727	Detected expression of Oct-4, Sox2 and Nanog in human endometrial adenocarcinoma samples
Götte et al. (2011) PMID: 20850729	Reported aberrant expression of pluripotency marker SOX-2 in endometriotic samples
Cervello et al. (2011) PMID: 21623195	Reviewed that most likely markers for endometrial somatic stem cells include Oct-4, Musashi-1, CD31, CD34, and CD144
Pachiarotti et al. (2011) PMID: 21075367	Reported nuclear OCT-4 and c-Kit expression in epithelial and stromal cells of endometriotic endometrium suggesting a stem cell origin of endometriosis. 10 folds higher and more intense nuclear OCT-4 expression in ectopic endometriotic tissue
Bentz et al. (2010) PMID: 20412569	OCT-4 expression was studied in human follicular (*n* = 49) and luteal (*n* = 40) phase endometrium. They detected OCT-4 mRNA and protein expression in all samples but did not find any differential expression across menstrual cycle
Forte et al. (2009) PMID: 19690622	Differential expression of stemness markers (SOX2, SOX15, ERAS, SAL4, OCT4, NANOG, UTF1, DPPA2, BMI1, GDF3, ZFP42, KLF4, TCL1) was reported in endometrial and endometriotic tissue by RT-PCR. OCT-4 was detected in all the samples studied
Matthai et al. (2006) PMID: 16421218	All human endometrial samples studied showed OCT-4 mRNA by RT-PCR and protein was expressed in the cytoplasm of few stromal cells

By transplanting GFP + SSEA-1 + *OCT-4* + stem cells into Swiss mice uterus during diestrus stage, we successfully delineated how the uterine stem cells participate during uterine remodeling under physiological conditions within the same estrus cycle. GFP + stem cells expressing SSEA-1 and *Oct-4A* differentiated into both LE and GE within 4 days in the same estrus cycle. These results provide better candidate stem cells that efficiently regenerate both LE and GE and are more primitive to AXIN2 + stem cells which took 20–22 estrus cycles to occupy the whole gland [1, 9]. True stem cells are expected to provide significant contribution during remodeling and our results suggest that OCT-4 positive VSELs/EnSCs are capable to achieve this.

Endometrial scratching is a technique that has excited infertility experts since 2003 when it was first advocated to improve success rate in ART clinics [39]. Various mechanisms have been suggested which could possibly result in improved success including (1) might improve stromal cell decidualization which increases the probability of embryo implantation (2) may induce aseptic inflammation and wound healing by secretion of inflammatory factors, such as cytokines, growth factors, interleukins, macrophages, and dendritic cells that could support embryo implantation and (3) may retard endometrial maturation, which is abnormally advanced due to controlled ovarian stimulation [40]. As evident from our results, uterine scratching led to increase in the numbers of OCT-4 positive stem cells in the endometrium. OCT-4 positive stem cells (VSELs with nuclear OCT-4 and EnSCs with cytoplasmic OCT-4) were distinctly visualized as small spherical, darkly stained cells with minimal cytoplasm in the basal region of luminal epithelium. They differentiated into epithelial cells and underwent  active cell divisions and also clonal expansion. We have earlier reported asymmetrical and symmetrical divisions and clonal expansion of uterine stem cells [13]. Similarly, Xiao et al. [41] reported increased expression of SOX-2, NANOG and OCT-4 in a mouse model of lipopolysaccharide-induced acute uterine injury and intrauterine adhesions by Western Blotting and qRT-PCR. Evidently, OCT-4 eventually gets degraded as cells differentiate further and is not expressed in mature epithelial cells.

These increased number of epithelial progenitors due to scratching differentiate into epithelial cells and provide a good cushion for implantation to occur. It is still not clear which population of infertile couples will benefit from endometrial scratching. We propose that infertile patients whose uterine stem cells are normal will respond positively to endometrial scratching and success rates will improve. But patients with dysfunctional uterine stem cells (due to various insults including perinatal exposure to endocrine disruption) will not show any improvements after endometrial scratching as reported recently in mice [16]. This could be the possible reason why recent Cochrane review [42, 43] painted a mixed picture regarding efficacy of uterine scratching in the Clinics and Hoogenhuijze et al. [44] suggested that endometrial scratching still should not be considered standard method of care.

OCT-4 positive stem/progenitor cells were also observed in large in adult endometrial sections due to neonatal exposure to endocrine disruption. This increased numbers of OCT-4 positive cells are suggestive of immature state of the endometrium and is associated with various uteropathies including non-receptive uterus, hyperplasia, cancer and adenomyosis [16, 45].

Our results challenge an earlier study where the authors expressed their concern on OCT-4 being reported in multiple adult cell types [46]. By doing careful knockout of OCT-4 in intestine, bone, hair follicles, brain and liver in mice, they observed normal development and function in these organs. Based on these results they concluded that OCT-4 is active in embryonic stem cells and not in adult mice tissues. They concluded that embryonic gene OCT-4 has no role in regenerating adult tissues. With greater understanding on OCT-4A expression in pluripotent VSELs in adult tissues since 2007, data in now published showing that these stem cells are easily mobilized to the organs where there is stress [15]. Most likely when OCT-4 was knocked out from the adult organs by Lengner et al. [46], OCT-4 expressing VSELs were mobilized to the affected tissue from other sites and restored homeostasis. Thus OCT-4 expression in adult tissues should not be looked at with disbelief.

Use of modern techniques like lineage tracing studies and scRNAseq fail to detect the rare population of quiescent VSELs. Present study provides evidence that OCT-4 positive stem/progenitor cells do exist in adult mice uterus and participate in endometrial regeneration under normal physiological conditions, after mechanical injury and after neonatal exposure to endocrine disruption. We have earlier discussed that the defective OCT-4 expressing epithelial progenitors are responsible for the initiation of various uteropathies in adult life [16].

## Conclusions

To conclude, VSELs are the true and elusive uterine stem cells that along with EnSCs comprise two populations of quiescent and actively dividing stem cells in the adult uterus. VSELs/EnSCS participate in regular endometrial regeneration and also get activated in large numbers upon endometrial injury and upon neonatal exposure to endocrine disruption. Dysregulation of VSELs/EnSCs results into various uteropathies in adult life including endometrial cancer.

### Significance of the study

Nuclear OCT-4 positive, small sized VSELs along with slightly bigger EnSCs with cytoplasmic OCT-4 exist in adult mice uteri. VSELs/EnSCs significantly contribute towards regeneration/remodeling of endometrial epithelial cells during estrus cycle. Improved endometrial thickness due to asymmetrical, symmetrical divisions and clonal expansion of stem cells after endometrial scratching helps improve outcome in infertility clinics. Further, neonatal exposure to endocrine disruption results in increased expression and dissemination of OCT-4 positive cells into myometrium. Similar increased OCT-4 expression is reported in various human uteropathies including endometriosis and cancer (Table 1) suggesting crucial role of VSELs/EnSCs in human endometrium and their dysregulation possibly initiates various pathologies. Human  endometrial VSELs will be small-sized cells which can be studied by flow cytometry with surface phenotype of LIN-CD45-CD133+ and will express OCT-4 and SSEA-4, besides other markers. 

## Supplementary Information

**Additional file 1.** Hematoxylin and Eosin stained images and earlier work on mice uterine VSELs.

## Data Availability

Supplementary section provides additional data.
