# Supplementary material for: Additional evidence to support OCT-4 positive VSELs and EnSCs as the elusive tissue-resident stem/progenitor cells in adult mice uterus
Source: Stem Cell Res Ther. 2022 Feb 5;13:60. doi: 10.1186/s13287-022-02703-8 (PMC8818151; doi:10.1186/s13287-022-02703-8)
Supplement: Supplementary file 1 — Additional file 1. Hematoxylin and Eosin stained images and earlier work on mice uterine VSELs. [file 13287_2022_2703_MOESM1_ESM.pdf]

SUPPLEMENTARY SECTION

Clinching evidence to support OCT-4 positive VSELs and EnSCs as the elusive tissue-resident stem/progenitor cells in mice uterus

Pushpa Singh, SM Metkari, Deepa Bhartiya, Stem Cell Biology Department, ICMR-National Institute for Research in Reproductive Health, Jehangir Merwanji Street, Parel, Mumbai 400 012, INDIA

Table S1: Published data on mice uterine VSELs/ EnSCs in mice uterus by our group

**Publications**	**Salient points**
Gunjal et al 2015	Two populations of stem cells were visualized in the uterine smears including small sized VSELs with nuclear OCT-4 and EnSCs with cytoplasmic OCT-4<br>They survive ovariectomy<br>VSELs specific transcripts vary in response to E, P, E+P and after 2 days of withdrawal of E+P
Bhartiya et al 2017	Reported VSELs in mice uterine myometrium
James et al 2018	Reported effect of treatment with E, P and FSH on VSELs/EnSCs in mice endometrium<br>Showed asymmetrical, symmetrical & clonal expansion of VSELs/EnSCS in mice uterus
Singh and Bhartiya 2021	Reported robust protocol to enriches stem/progenitors' cells (VSELs/EnSCs). VSELs express OCT-4, NANOG, SCA-1, SSEA-1, c-KIT and were studied by flow cytometry (LIN-CD45-SCA-1+) and qRT-PCR (*Oct-4A, Sox2, Nanog, Stella, Fragilis*). Flow cytometry data shows maximal numbers of VSELs during estrus and metestrus phases. OCT-4 positive VSELs/EnSCs co-expressed ERα, ERβ, PR and FSHR and thus are expected to be directly regulated by ovarian hormones and also become vulnerable to endocrine disruption.
Singh and Bhartiya 2021	Study reveals stem/ progenitors' cells upregulated after neonatal exposure to endocrine disruption, shows Erβ dominance and progesterone resistant phenomena and their role in initiating various uteropathies independent of the circulatory hormones in adult life.
Singh and Bhartiya 2021	VSELs/ EnSCs were highly upregulated after neonatal exposure to endocrine disruption. OCT-4 positive cells co-expressed with cancer stem cell marker CD166 & ALDH1A suggesting pluripotent stem cell acquire cancer stem cell property after neonatal endocrine disruption and initiate oncogenic events and various endometrial pathology

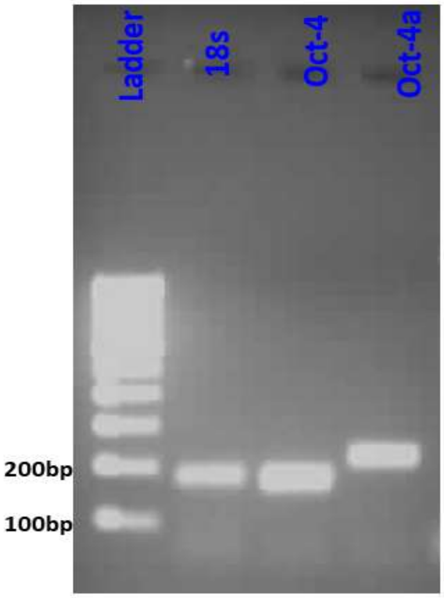

Fig S1: RT-PCR results showing expression of transcripts specific for *Oct-4* & *Oct-4a* on GFP+SSEA-1+ stem cells. Both the bands appear to be of similar intensity suggesting it comprises mainly of VSELs. In normal intact tissues, transcript specific for Oct-4 is predominant while Oct-4a is minimally expressed.

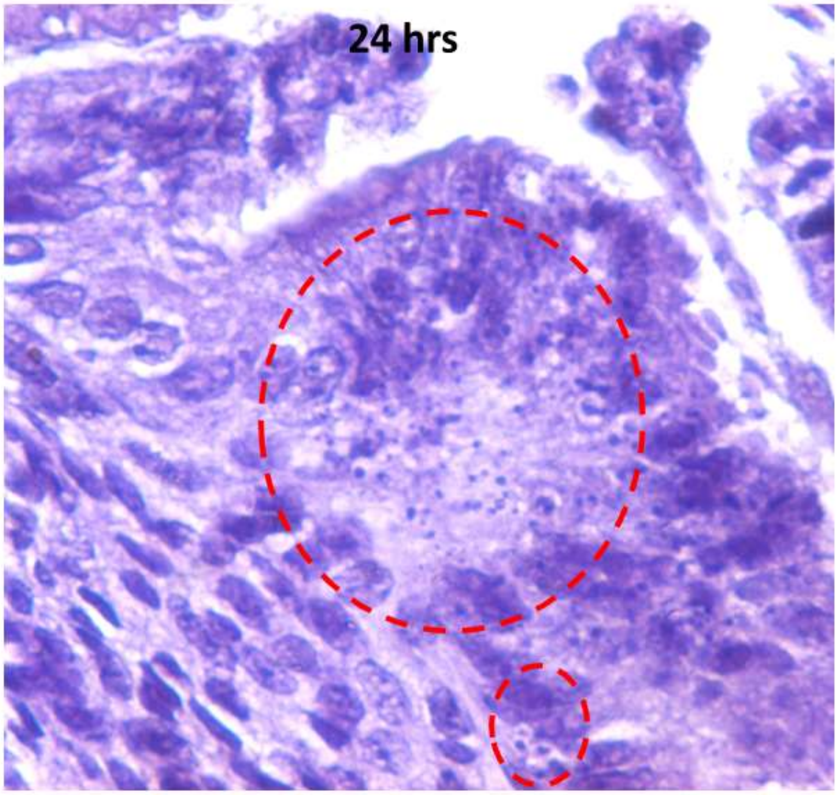

Fig S2: Note the presence of small-sized spherical cells (red broken circle) with dark-stained nuclei and high nucleo-cytoplasmic ratio after 24 h of scratching. These are the putative VSELs that increase in numbers in response to endometrial scratching. They appear to be of variable sizes since they are cut in different planes of section. Similar stem cells were also reported earlier (Singh and Bhartiya 2021b, James et al 2018).

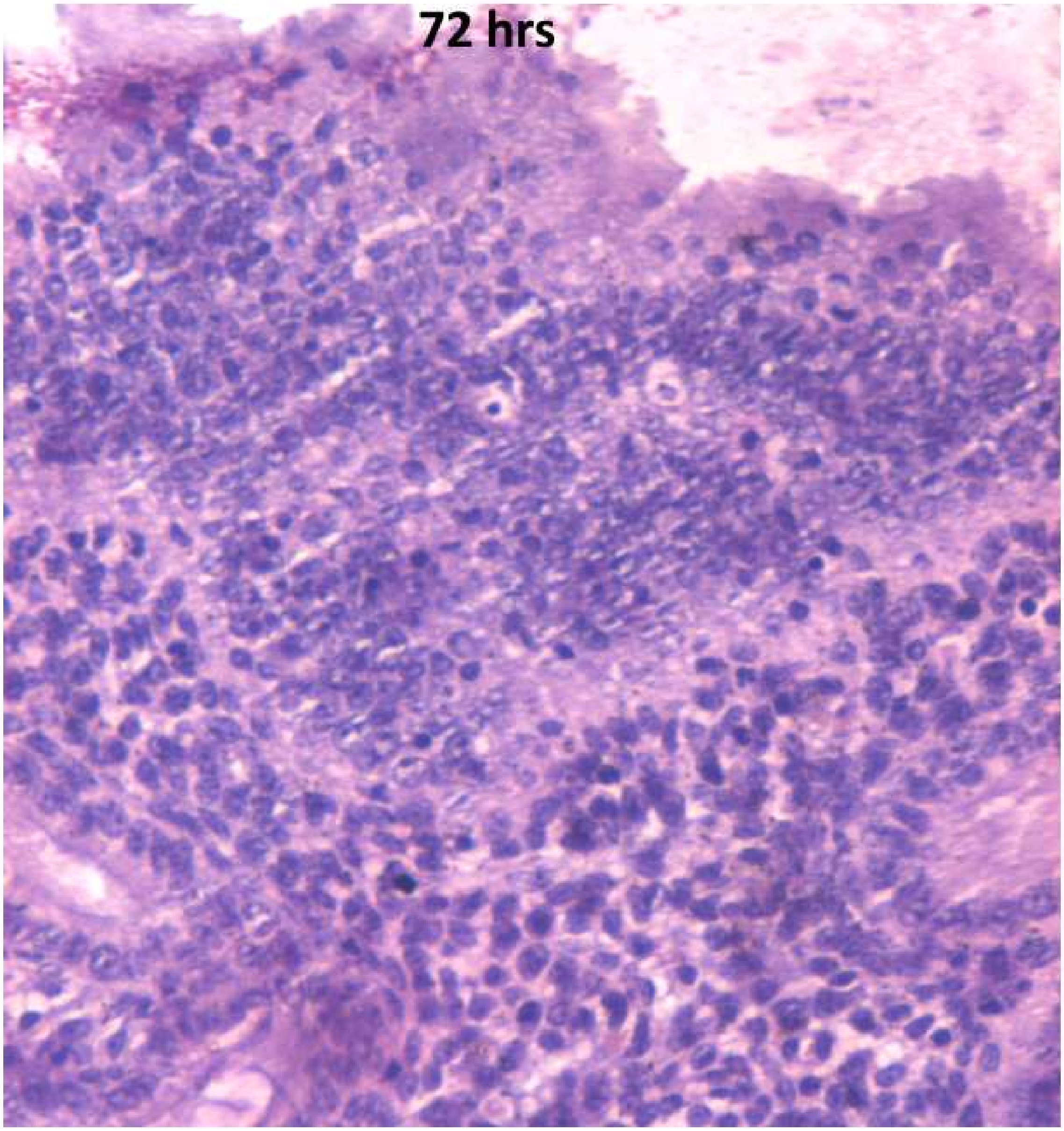

Fig S3: Rapid expansion of epithelial cells which evidently differentiate from the stem cells by 72 h.

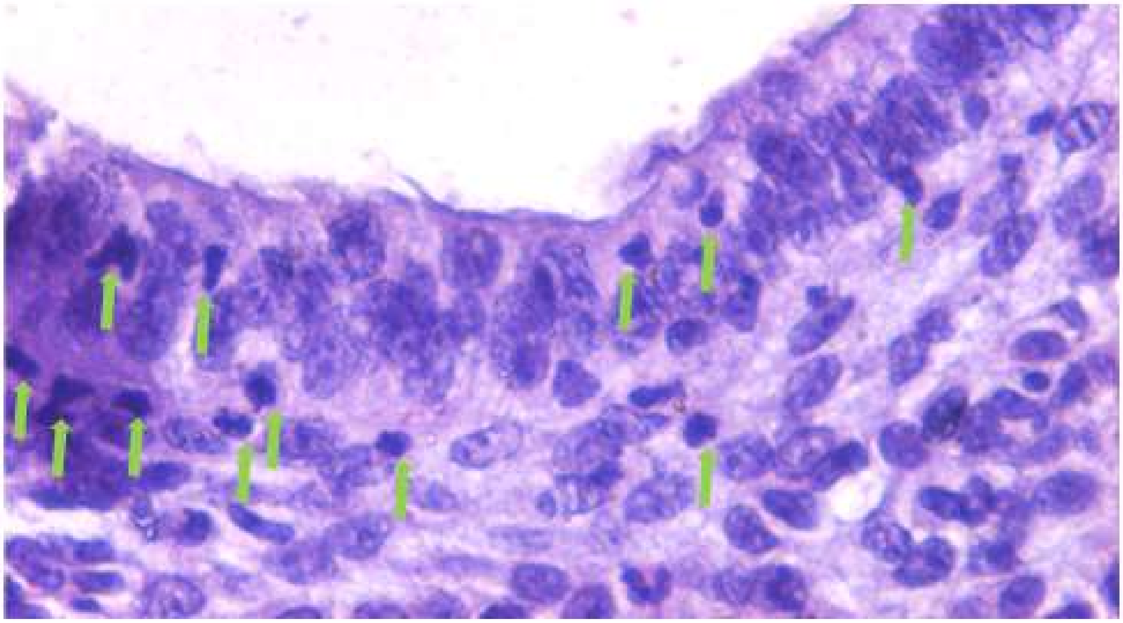

Fig S4: Increased proliferation of epithelial cells by the presence of mitotic figures (green arrows)

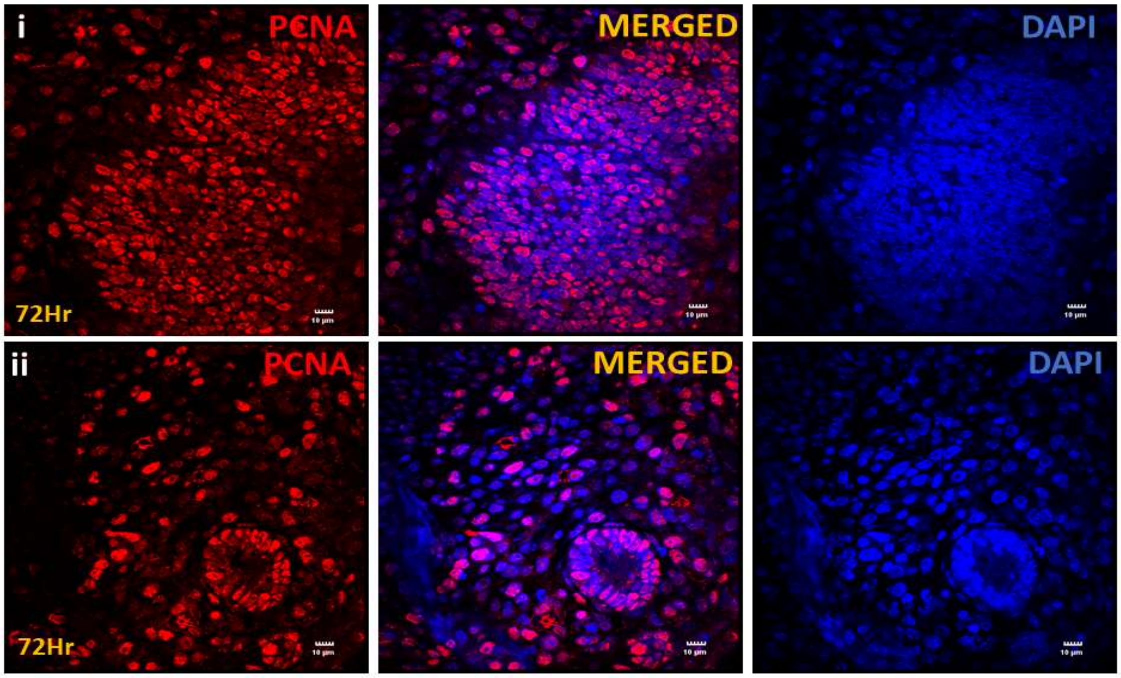

Fig S5: PCNA expression in endometrial sections after 72 h of uterine injury. Large numbers of PCNA positive progenitor cells are evident in both the (i) luminal and (ii) glandular epithelium.

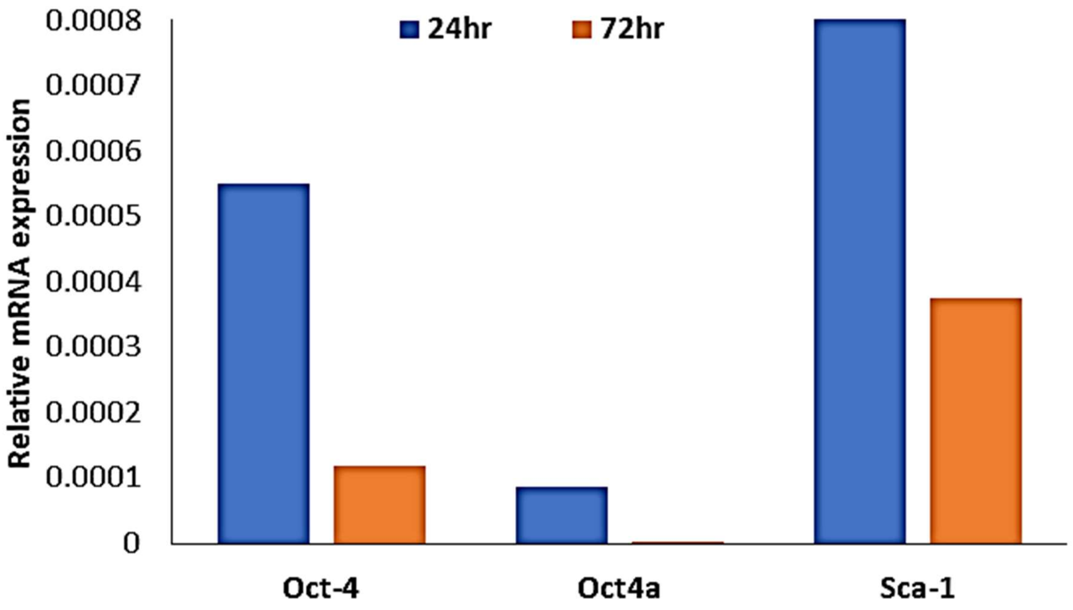

Fig S6: Relative mRNA expression shows higher expression of transcripts specific for pluripotent and stem cell markers (Oct-4, Oct4a, Sca-1) after 24 and 72 h of uterine injury. As evident, VSELs and EnSCs expressing Oct-4A, Oct-4 and Sca-1 are increased by 24 h and their numbers decrease by 72 h.
